# 14th Annual University of Pennsylvania Conference on statistical issues in clinical trials/subgroup analysis in clinical trials: Opportunities and challenges (morning panel discussion)

**DOI:** 10.1177/17407745231175078

**Published:** 2023-06-16

**Authors:** Kit Roes, Janet Wittes

**Affiliations:** 1 Department of Health Evidence, Radboud University Medical Center, Radboud University, Nijmegen, The Netherlands; 2 Wittes LLC, Washington, DC, USA

Jim Lewis: It is my pleasure to introduce our first panelist, Dr Janet Wittes. She is an elected fellow of the American Statistical Association, the Society for Clinical Trials, the American Association for the Advancement of Science, as well as the International Statistical Institute.

Janet T. Wittes: I want to start with a graph summarizing subgroups from the Systolic Hypertension in the Elderly Program, a trial that randomized 4736 elderly participants.^1^ This graph (Figure 1), prepared by Professor Robert Byington, shows the effect in subgroups of blood pressure lowering on fatal and non-fatal stroke in people with isolated systolic hypertension. For every categorical variable, Dr Byington looked at the subgroups defined by the specific categories. In addition, he dichotomized every continuous variable at the median to produce two subgroups. He then looked at the estimated effect sizes in each of these many subgroups.

**Figure 1.**
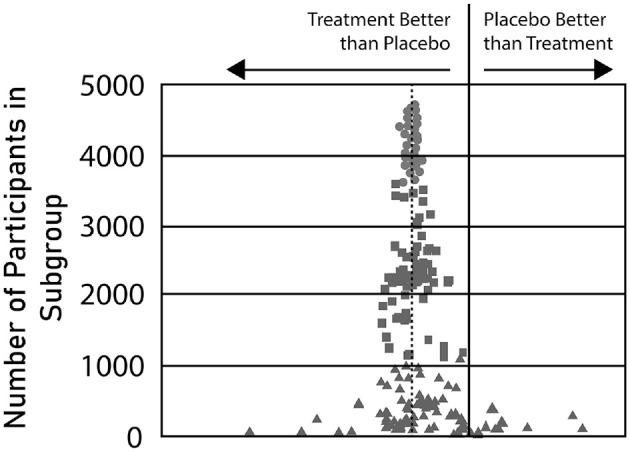
Systolic hypertension in the elderly trial (SHEP)—relative risk of fatal and non-fatal stroke. The circles represent the large subgroups (size above 3500); the squares represent the middle-sized subgroups (size between 1000 and 3500); and the triangles represent the subgroups of size below 1000. The x-axis is relative risk with the solid vertical line representing a relative risk of 1 and the dotted line a relative risk of 0.7. Because the overall relative risk in the SHEP trial was approximately 0.7, the dots center around the dotted line.

What you see is perfectly consistent with what we have heard from Drs Fleming, Kent, and Unger this morning. The top of the graph shows the subgroup(s) that include a large percentage of the participants; in those, the estimated effect size naturally clusters around the overall effect size. The squares are the middle-sized subgroups. These cases, although they show some variability in estimated effect, generally tell nearly the same story as the large subgroups do.

Where you begin to see results that are not believable are the estimates shown by the triangles; these represent the very small subgroups. If you believe the triangles on the right, you would conclude that in some identifiable subgroups lowering blood pressure *increases* stroke risk considerably. However, if you take the triangles on the far left seriously, you would have to believe that in other identifiable subgroups lowering blood pressure dramatically reduces the risk of stroke. Obviously, this huge variability reflects noise.

This next case echoes some of Dr Fleming’s examples. The trial took place in the early part of this new century. A paper published in 2001 reported results from a randomized controlled trial to test whether an experimental monoclonal antibody, oregovomab, increased survival time in women with ovarian cancer.^2^ The data showed that the proportion of high-risk patients who experienced disease-free survival at 6 months was 79% in those randomized to oregovomab and 39% in those randomized to placebo (*p* = 0.0397) (As an aside, I cannot understand the practice of citing over-precise *p*-values. Why not say 0.04?).

Unstated in the paper was that the data included only half of the participants: overall, the data showed no effect of the drug. Indeed, in the lower-risk group, those randomized to drug had *higher* disease-free mortality than those on placebo. At the time these data emerged, it seemed to me that if the investigators were willing to believe the high-risk group experienced benefit, they also had to believe that the low-risk group was harmed. Or at least they had to acknowledge that the evidence was just as strong for harm in the low-risk group as it was for benefit in the high. A more parsimonious explanation was that the data overall showed evidence of neither benefit nor harm. But the investigators went ahead and carried out a Phase 3 trial in the high-risk group, which is different from what the investigators did in the Duke trial that Dr Fleming described. The Phase 3 trial showed no evidence of a difference between the active and placebo groups on disease-free survival.^3^ This is a cautionary example of acting on the data from a subgroup in an early phase trial.

Typical forest plots reflect some of the points that all three speakers, especially Dr Kent, discussed: forest plots show one-at-a-time variables. If I, a woman over age 75 years, look at a forest plot describing results from a trial, I show up in different subgroups. I show up in the subgroup of women. I also show up in the “greater than 75 age” subgroup. Depending on which subgroup I think is most important for me, my treatment may differ. Surely, this is not how we should be looking at data. My own view, which a group of us expressed in a paper we wrote in 1991, is to apply statistical methods that capture the framework of the prior hypotheses.^4^ And in light of Dr Kent’s warning, avoid one-at-a-time subgroup inference. Place greater emphasis in the overall result than what may be apparent within a particular subgroup.

Another important principle: distinguish between prior and data-derived hypotheses. As Dr Unger warned, don’t have too many prior hypotheses. My friend Professor David Zucker cautions that pre-specifying a host of hypotheses is equivalent to having nothing pre-specified. Still another recommendation: use tests of interactions or correct for multiplicity of statistical comparisons or do both. If you proceed in that manner, often your conclusion would be, “I have looked at lots of subgroups. The estimated effects appear to differ but when I correct for multiplicity or test for interaction, I see no convincing evidence of differences by subgroup.”

While I am extremely cautious about making inferences from subgroups, over time I have developed an evolving worry that some types of subgroups are in fact important. Dr Kent mentioned that important risk factors may define relevant subgroups. As Dr Unger mentioned, regulators are interested in geographic region because standard of care, diet, and risk factors may differ markedly from country to country.^5^ In large global trials, China often enters a few years after the trial begins. In trials of a drug with a delayed effect, for example, cardiovascular trials of lipid-altering intervention where the outcome measure is hazard ratio calculated assuming proportional hazards, the late entry of China may lead to an attenuation of estimated effect. Another example is the reporting of adverse events. In my experience, many trials show very large differential reporting of serious adverse events from country to country, with Russia often showing much lower rates than other countries.

Then there’s the TOPCAT study of the effect of spironolactone in class 2 heart failure. In that study, Russia and Georgia showed no effect of drug, while data from the other countries combined showed a magnitude of effect very close to what had been pre-specified. A paper by Pfeffer et al.^6^ carefully discusses this finding.

Recent thinking has led to more sophistication about subgroups. Dr McShane talked about personalized medicine, especially biomarker-based subgroups, the subgroups we are likely to deal with in the future. In our paper way back in 1991,^4^ we urged interpreting results in the context of similar data from other trials, from the “architecture” of the entire set of data on all patients, and from principles of biological coherence. Those are some of the methods we heard from Dr McShane. She warned us that even elegant methods of looking at biomarkers or biomarker-based subgroups suffer from all kinds of problems. For example, there may be tumor-specific or assay-specific differences. She pointed out the need to have a logical basis to choose cut-points for biomarkers. Her examples also show one of the problems with using hierarchical methods for looking at subgroups.

We have much to learn about how to capitalize on these new methods of personalized medicine. I thank the speakers for very interesting, provocative talks. I look forward to this afternoon as we hear about other ways to think about looking at subgroups.

Jim Lewis: Thank you so much. It’s now my pleasure to invite Dr Kit Roes to join. Dr Roes is joining us from the Netherlands, where he is Professor of Biostatistics at Radboud University Medical Center and a visiting professor of Clinical Trial Methodology in Utrecht. He has done a great deal of research on methodology of clinical trials, and we are very interested to hear his thoughts this morning.

Kit Roes: Thank you very much for the opportunity to participate. Besides being a professor, I’m also involved in biostatistical work in drug regulatory decision-making for the European Medicines Agency, the European Union agency responsible for the evaluation and supervision of medicines. I will give you a summary view of the European regulatory thinking on subgroup analysis and confirmatory trials, and then give some reflections on the truly interesting presentations of this morning.

From a regulatory perspective, I always tend to be modest when we authorize a new drug. We essentially add a new drug to the armamentarium of treatments available to physicians and we provide the best information possible. But in the end, it’s the physician who needs to make an evidenced-based decision to treat the next patient. I still experience quite a gap between approving a drug and being able to provide the information that truly covers the evidence the physician needs for such decision-making. And quite a bit of that gap is the lack of understanding of the variability of treatment effects in subgroups.

I admit that, for most statisticians, the following is a simplified view. If there is a treatment effect, you can hold two possible beliefs: one it’s equal for all subjects, which is in statistical terms, strong additivity; or if there’s a treatment effect, it will likely vary among subjects. I think that probably no one in the audience will go for the first. This means that in regulatory assessments approving new drugs to add to the treatment options, despite all the caveats, exploring effects for relevant subgroups, is essential in assessing benefit/risk.

I think it’s good to understand the European regulatory decision-making: a positive decision requires the benefit/risk to be positive, across the targeted populations. So, exploring subgroups is truly essential, and despite the caveats, regulatory decisions are determined while also relying on subgroup analyses in confirmatory trials. A guideline articulating our thinking and practice regarding subgroup analysis was recently published.^7^

Roughly, those regulatory decisions fall into the following three categories of evidence:

- The conclusions of therapeutic efficacy (and safety) apply consistently across subgroups of the clinical trial population.
- The benefit-risk is borderline or unconvincing: identify post hoc a subgroup, where efficacy and risk-benefit is convincing.
- The clinical data fail to establish statistically persuasive evidence: identify a subgroup, where a relevant treatment effect and compelling evidence of a favorable benefit–risk profile can be assessed.

The first is the simplest one, where the overall result is truly convincing across subgroups. Second, and more complicated, is the case where the benefits/risks are borderline or unconvincing, although proven according to the usual standards of type 1 error control. Then, subgroup analyses can aim to identify, post hoc, subgroups where efficacy and benefit/risk are convincing, that thus the efficacy does outbalance the additional safety burden of the patients. The third situation is the most challenging one, where the clinical data failed to establish statistically persuasive evidence, so it did not meet standards. But given the urgent need in the particular setting in the disease, it might still be relevant if you could identify a subgroup where both the relative treatment effects are convincing and the evidence is compelling enough for benefit/risk to be positive in that subgroup. No doubt, this is the most complicated setting where we need to be extra careful.

Just to give you an idea how often these scenarios occur in regulatory assessments, we reviewed assessments of 162 products of which 138 were approved. We evaluated the extent to which “Major Objections” or “Other Concerns” were related to subgroups. A substantial number of products had a major objection related to subgroups, which, if not resolved, would be a reason for refusal and not providing the license.

It happens often in regulatory assessments that decision-making does include subgroup assessment. The types of problems are illustrated in the two example quotes from assessment reports (Figure 2), where the problems identified are not based on statistical significance alone.

**Figure 2.**
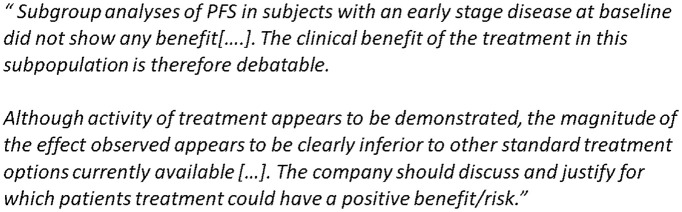
Quotes from assessment reports.

What we have tried to do in the guideline aimed to directly support the assessment process. We created two flow diagrams and distinguished the simple and more complicated situations as a decision tree where in the end there is an assessment of the level of credibility of the subgroup results in a confirmatory trial. So, credibility is one of the key concepts. And as we’ve heard, all of the speakers spoke to key elements in terms of level of evidence, the a priori definition of any subgroup analysis, and the biological plausibility of a particular finding. Of course, replication is also fundamental and can also exist “in parallel.” If there are multiple independent data sources where the same subgroup effect is observed, that can hold as a replication and adds to the credibility of a subgroup finding.

This is a somewhat high-level overview of the regulatory thinking. I will now provide reflections on the interesting presentations, and what I take away from them. And I would suggest that it’s not a full reflection; rather I address the points I would like to highlight for discussion.

I think the important point made by Dr Kent is to explore clinically relevant subgroups rather than looking at a standard set because we are used to it (e.g. age and gender). We look at the risk, measured in terms of the primary outcome, but also at outcomes that are really reflective of actual risk and on factors that are risks for adverse outcomes. For example, I think age is usually not meant to be used as age, but as an indication of frailty, and frailty is a risk of a poor outcome.

I agree that most subgroup results are false or overestimated, and that this is especially a risk for small subgroups. Statistically, it’s important to acknowledge, and you can think of statistically smart estimation methods to correct for this issue. We have shrinkage estimation of effects in our toolkit, but it can actually be mathematically proven that it is impossible to solve this problem universally across the subgroup parameter space.

There’s an important paper following the original from Ioannidis^8^ that is subtitled with “But a little replication goes a long way.”^9^ So, I think we should really not forget about replication in any setting. I actually agree with Dr Unger; I think most subgroup analyses are in the end exploratory. In the regulatory sense, they are a part of secondary assessments. I would suggest a discussion of a more general interpretation of pre-definition. I do not necessarily expect pre-definition to be always formalized in terms of type 1 error-controlled hypotheses. I think what we would like to promote is that pre-definition of the biological rationale for expecting differential efficacy is essential, including the direction of expected effects. That is not at all common practice today.

This biological rationale and the expected directional effect of the difference of effects is crucial, because in my experience, any association has a plausible post hoc explanation. And if you present the reverse direction, you will also likely get a plausible explanation. I therefore do think that pre-definition of the biological rationale is going to help and does add credibility to any subgroup analysis.

Now, some reflection on the presentation by Dr Fleming. The significant role that subgroup analysis in the development of a particular drug candidate plays is not so very statistical, but it’s very relevant. From my long experience, it’s a huge factor in research that already starts at the exploratory development design state. I’ve seen multiple examples where a subgroup gets explored based on exploratory trials and people are disappointed when the subsequent trials fail, which I think is potentially a huge research waste.

I think we need to address required standards and decision criteria to pursue new trials in subgroups. I think Dr Fleming does hint at this, but I think we should be more explicit about what’s needed to set the next step in case of potentially relevant subgroup findings. While there is some work done on this, I think we need to step up there.

And finally, the presentation by Dr McShane stressed that we need to extend our ideas about statistical approaches for subgroup analysis. One can even wonder whether it’s subgroup analyses that we’re looking at with continuously measured biomarkers as a driver. We need to consider the biological rationale for biomarker assays and cut-off values, but also all the variance components that come to play. There’s variation in biomarkers within and between subjects, but also over time within and between subjects and these become essentially part of the concept of subgroups.

So, we need to rethink whether we actually have subgroups. I think we need appropriate modeling across the continuum. I would also like to support Dr McShane’s doubt about hierarchical testing. I note that not all my colleagues are in agreement here, but I see lots of hierarchical testing schemes which do not help me in making a good biological interpretation of the data. I would really promote being careful about focusing on what we can biologically understand.

David Kent: I enjoyed everybody’s talks quite a lot. It seems that there is a lot of convergence of our ideas. Perhaps it’s a result of us all having done these analyses for a long time and made many mistakes over many decades that we all realize how misleading these subgroup analyses can be.

In terms of judging the credibility of subgroup analyses, I just want to make sure everybody is aware there is an available tool that has formalized how to judge the credibility of subgroup analyses. It’s called ICEMAN^10^ developed by the group at McMaster. I think it’s a pretty good tool and uses criteria very similar to those that Dr Roes described. Basically, it relates the credibility to: (1) the strength of the prior evidence and (2) how strong the subgroup effect is, as reflected in the *p-value*. There are other criteria as well, that represent good practice, such as whether the subgroup was pre-specified, including the direction of the effect. There’s a total of five criteria that the group has recommended to be used to score every subgroup when performing a systematic review.

An issue that comes up again and again is how different patients in the same trial can be in terms of their outcome risk; you’ll have groups of patients who have a 1% risk of the outcome and groups of patients who have a 10% risk, and when you see that, you know the clinically relevant treatment effect, which is measured on the absolute scale, is going to differ in those two groups. You almost know that from the beginning, even if there’s no statistical test showing that the contrast in the relative effects is different.

From my perspective as a clinician, is often I’ll want to treat those patients quite differently. Dr McShane noted that oncologists frequently distinguish between prognostic markers (which predict outcomes) and predictive markers (which predict treatment response). But often the prognostic markers themselves are predictive on a different scale, the absolute scale; they’re effect modifiers on the absolute scale. So, we use a different nomenclature, but the distinction between predictive and prognostic markers is not as clear as some have suggested.

Lisa McShane: I’d like to thank the other speakers and the discussants. I thought it was a wonderful set of talks and I appreciate the really insightful comments from the discussants. I’d like to touch on a few points. First, I agree with Dr Kent’s last comment about the distinction between prognostic and predictive markers. Many of our genomic signatures that are used for determining whether a patient with early stage hormone-receptor-positive breast cancer should get adjuvant chemotherapy started out as prognostic biomarkers or signatures.

Even if there is a common relative risk (or hazard ratio) associated with treatment benefit across levels of a prognostic biomarker (or risk score), that relative risk would differ in terms of absolute risk reduction when you’re comparing chemotherapy versus no chemotherapy. I think that it is a distinction that often gets very muddied. But I do think we shouldn’t automatically assume that any prognostic marker will identify a group that is more likely to benefit from therapy, because in fact, that prognostic effect could mean that the patients who tend to have really bad outcomes (who we might think will benefit most from more, or alternative, treatment) are the ones who are just going to be resistant to every kind of treatment. So, we have to be cautious. I agree, it can be very confusing and our terminology is terrible. I know Dr Fleming always cringes when he hears the word “predictive” because it can have so many different meanings. “Treatment effect modifier” would be more appropriate.

I do want to comment also on Dr Roes’ comment about biological understanding in modeling. I agree, that sounds very attractive—although, I have to say in practice it’s been much harder than it sounds. I give as an example all the trials that have been done (typically in oncology it’s going to be single arm phase two trials with response rate endpoint) based on what seems to be strong biology and mechanistic rationale. In oncology, we have had these amazing successes with a few targeted therapies in subgroups of patients whose tumors have certain targets, for example, molecular alterations. This seemed like it was the answer.

So, we launched all kinds of trials of this type involving targeted therapies. And, I have to say that the results have been disappointing. You would think, if you have a mutation in a certain gene and you deliver a therapy that targets that, how can that not work? But in fact, although I don’t know what the exact rate of successful phase two single arm trials has been for targeted therapies, it’s probably somewhere around 20% or less of those trials. So, all this wonderful biology can sound great, but as with any prior beliefs, there is subjectivity. It all sounded perfectly coherent scientifically, but it just didn’t pan out.

The other challenge we face in oncology is the tumor-type heterogeneity. Many of these trials are conducted in multi-tumor cohorts, and things can vary a lot by tumor type. One of our phase two single arm trials accrued around 35 patients, and they encompassed 17 different tumor types. So, where do you start modeling and what assumptions do you make across, what at the beginning, would be even an unknown *number* of tumor types. It is very challenging, but I still believe that’s the direction we have to go.

Kit Roes: I see a problem in the types of trials that are being done from which we derive the information, as well as big challenges in modeling the efficacy. We still tend to create subgroups by dichotomizing markers using cut-off values. It’s a very strange idea that the patient belongs to a different subgroup just because his biomarker is one point smaller. That, of course, is not true. I think at that level, we need to be conscious about our statistical approaches.

Lisa McShane: Yes, I agree.

Kit Roes: So, I was thinking that’s a simple concept to be aware of—that we sometimes fall in the trap of always thinking in subgroups when it’s probably not a subgroup issue but something else that we need to describe.

Lisa McShane: Right, and also the multi-variant nature of things. Maybe you need to have two genes that are turned off to have the drug work and you quickly get into the high-dimensionality problem.

Tom Fleming: Drs Kent, Unger, McShane, Roes and Wittes make great points. I’d like to amplify some of their insights, starting with the criteria that are of integral importance for credibility of results in subgroup analyses: being statistically compelling, where pre-specification and independent replication are key and being biologically plausible.

Dr Unger properly emphasized the considerable importance of pre-specification. When I hear people say, “it’s pre-specified,” they often mean that a given covariate was included in the list of about a dozen baseline factors provided in the Statistical Analysis Plan (SAP) for performing analyses in about 25 subgroups. It is important to include such a list in the SAP because, if we see a beneficial effect, typically we want to understand whether the results are generalizable to the entire cohort eligible for randomization. However, such pre-specification does not establish the reliability of exploratory subgroup analyses conducted when the pre-specified primary analyses in a trial are not definitively positive.

Dr Unger also properly indicated that a particularly important element of pre-specification is whether subgroup analyses for a certain baseline factor are part of the protocol’s specification for alpha spending. In contrast, if a covariate is simply among a list of baseline factors specified in a later section of the SAP that addresses subgroup analyses, corresponding results usually would best be presented descriptively, typically using forest plots.

In addition to pre-specification, independent replication also is of considerable importance to statistical reliability of subgroup analyses, as indicated by Dr Roes. Pre-specification matters, not only when interpreting *p*-values but also in the interpretation of assertions about biological plausibility. One usually could obtain a *p*-value less than a targeted threshold for positivity, even for ineffective agents, simply by engaging a statistician to conduct a large number of analyses, and often this effort would include performing many subgroup analyses. If that “favorable”*p*-value arose from an exploratory subgroup analysis, then clinicians, with their breadth of clinical insights, should be able to provide some justification for the biological plausibility of effect modification by that baseline covariate.

Justification for biological plausibility is much more persuasive if formulated by those who do not have access to trial results rather than by those who have been shown the results and then asked to provide a biological explanation. For example, if the trial unexpectedly reveals that the beneficial effects were seen mainly in females, rather than sharing this result with clinicians and asking for biological explanations, these clinicians should be asked before seeing trial results to provide a summary of the baseline covariates that would be the most plausible effect modifiers. In turn, an important role of the SAP is to provide pre-specification of potential meaningful effect modifiers, if there is considerable prior evidence that one or more baseline covariates would meaningfully influence level of treatment effect.

Another issue of considerable importance was raised by Dr McShane in her presentation, and then illustrated in the analysis of the Checkmate 649 Trial.^11^ As in that trial, suppose there is sufficient biological evidence at trial design that treatment effect would be stronger in biomarker positive relative to biomarker negative patients such that, hierarchically, alpha would be spent first in the biomarker positive subgroup and, if statistical significance is achieved, then treatment effect would be assessed in the pooled sample of biomarker positive and biomarker negative patients. While this approach properly adjusts for multiplicity of testing, it is logically inconsistent to exclude the biomarker negative patients when assessing effects in the biomarker positive patients, yet then allow the positives to meaningfully influence the assessment of effect in the negatives. As argued in the Rothmann et al.^12^ article referenced by Lisa McShane, once efficacy has been established in the biomarker positive patients, extrapolating a conclusion of benefit to the biomarker negative patients as well should require not only positivity in the pooled analysis but also that the estimated effect in the biomarker negative patients is of sufficient size both to be clinically relevant as well as to yield statistical significance in an analysis of the pooled sample if this were the common effect size in that pooled sample.

Jim Lewis: Many of you made the point today that we shouldn’t report *p-*values for subgroup analyses, and there was also some discussion about using a different nomenclature in reporting them. But aren’t we talking about treatment effect heterogeneity? I wonder if there are thoughts about enhancing forest plots with confidence intervals, as well as providing a *p-*value for the test of heterogeneity?

Tom Fleming: Being a frequentist, I have a strong interest in protecting the interpretability of *p*-values. As indicated in my presentation, for a proper interpretation of a *p*-value, we need to understand the sampling context. This reasoning also applies to the interpretability of an array of *p-*values from interaction tests.

There are additional challenges when using tests for interaction to assess effects in subgroups. Since tests for interaction are inherently underpowered, lack of statistically persuasive evidence of heterogeneity doesn’t mean that clinically meaningful true heterogeneity doesn’t exist. Consistent with insights from Dr Unger, the most reliable and interpretable results are those provided by the pre-specified alpha-spending analyses. While it is useful to consider results in exploratory subgroups, these should be presented descriptively, such as through use of forest plots. Please put away the corresponding *p*-values.

False conclusions of lack of benefit could also arise when using tests for interaction. In particular, even if a test for interaction by a baseline covariate was statistically significant, treatment could provide important benefit in all patients. It could work fantastically well in the positives and adequately well in the negatives, and yet there could be significant interaction. Hence, reliable evidence about effects in subgroups requires richer insights than what would be provided by tests for interaction.

David Kent: If I can pick up on some of these things that Dr Fleming just introduced, we should distinguish in our own minds what the purpose of these *p*-values is. As he just said, they don’t really give us the information that I as a doctor want. I want to know whether there are clinically important heterogeneous treatment effects, which basically has to be evaluated on the absolute scale, and it means that the treatment effects on the absolute scale span some critical threshold—such that some patients should be treated one way and others another.

Whereas, what we’re testing with a *p*-value is generally a contrast in *relative* effects. And I’m not sure what people take away from these contrasts in relative effects, but they probably think about it mechanistically, even though they don’t really show biological interaction, because the variable being examined, the subgrouping variable, might just be a proxy for other variables that are not known.

If we look at males versus females, old versus young, those with hypertension versus those without, and we find a significant *p-*value for interaction, it doesn’t mean that that is mechanistically what’s modifying the effect, because it can just be some unobserved third variable that’s correlated with the subgrouping variable. Nevertheless, knowing the biological mechanism is generally not strictly needed for targeting therapy.

Jim Lewis: Yes, I agree.

David Kent: So, we have to be careful of what conclusions we draw with those *p-*values. They’re not that clinically informative, nor are they necessarily mechanistically informative. And finally, even when they are significant, we tend to overvalue or overinterpret them or basically, underestimate the likelihood of false positives. Because as Dr Fleming and others have said, these are underpowered analyses. When we think of underpowered analyses, we think of that as a problem with the sensitivity of the analysis, but it’s also a problem with the false positive rate, because as your power goes down, your false positive rate shoots up. That’s the environment that we’re working on with subgroups.

Jim Lewis: It will be great to hear comments from the speakers about using (pre-specified or not) data-driven approaches, such as machine learning, to identify subgroups, including latent variables.

David Kent: In low power environments, machine learning approaches using many covariates carry with them many of the same risks of these one-variable-at-a-time subgroup analyses—although there are honest ways to hold out data and retest. But until we start seeing replication and reliable results across several different domains, I will probably remain skeptical as to how frequently these machine learning approaches can really contribute.

I think we are so far from having the amount of statistical power we would need to have informative machine learning approaches to find these subgroups. You may need not a 10-fold, but maybe 100-fold sample size when you’re looking at a lot of variables than you have in a typical trial powered to find a main effect. So, I think with the sample sizes we have, we are unlikely to see a significant contribution from agnostic machine learning approaches.

Tom Fleming: To add to what Dr Kent is saying, it is important to distinguish between “predictive” and “prognostic” covariates. When treatment effect differs by levels of a baseline covariate, Dr McShane is right that I prefer to refer to these covariates as having meaningful treatment by covariate interaction rather than as “predictive” covariates because a dictionary frequently indicates that the word “predictive” means “prognostic.”

A baseline covariate is “prognostic” if it is associated with the trial outcome in natural history settings or, more specifically, in participants receiving the control arm of the trial. Hence, given the breadth of this context, one might justify potential utility for machine learning approaches to provide further enlightenment in identifying prognostic covariates.

In contrast, whether a baseline factor is a “predictive” covariate, (i.e. has meaningful treatment by covariate interaction), is specific to the effects of the experimental intervention or its class of interventions. Given the inherently more limited amount of such information, it is less clear what the upside utility of machine learning approaches would be for identifying predictive covariates. Nevertheless, I still am interested to learn about these issues from colleagues in the afternoon presentations.

Lisa McShane: If I can chime in a little bit, this is one of my favorite topics, and I agree with what Drs Fleming and Kent have said. First, I think we’re often unrealistic about the sample sizes required to have any chance of developing a reliable classifier or predictor from high-dimensional data. You know, they work great if you’re trying to predict which customers on the Amazon website are going to buy your product if you flash an advertisement on their screen, but that’s a totally different ballgame than what we’re talking about.

We have different scenarios. We *might* have a few thousand patients but that’s fairly unusual. We typically have a few hundred patients in the best scenarios. Our data dimensionality may be 10 or even 100 times that, so it’s really challenging. And the other issue that Dr Fleming alluded to is that many of these predictors have been developed—I’ll call them the high-dimensional data predictors—emerged from analyses in the mode of “can you throw in the numbers and come up with something that associates with outcome?” The kind of approach is completely devoid of consideration of what it is you’re trying to do with that predictor. Is it prognostic? Is it for treatment selection? Are you using a heterogeneous group of patients who make no sense clinically to look at together?

There are huge issues. That said, there are people who know what they’re doing with machine learning methods. They know how to do it properly to make sure that they don’t overfit models. I don’t want to diminish the efforts of people who are doing that. But it’s very challenging with the balance of sample size to dimensionality that we have in most of our studies.

Mark Rothmann: We are currently revising our earlier gender differences/sex differences guidance. We should always look for differences in treatment effects between males and females in studies and investigate any apparent difference that was not anticipated beforehand.

Now if there hasn’t been a history of differences, reproducibility of demonstrating a difference in treatment effects is important. And we have seen differences between males and females where it’s been due to age with the females being older. We have seen differences where weight or body mass index (BMI) seems to matter and the distribution of weight or BMI is different between the sexes. We’ve seen studies oversized for effectiveness, sized for safety—for example, in first approval of cholesterol-lowing products. A statistically significant and clear interaction effect by sex was present with males getting a larger additional average decrease in the percent change in cholesterol than females. But we don’t know if this translates into any difference between males and females in cardiovascular risk reduction.

How would a Food and Drug Administration (FDA) reviewer respond to such finding? I think we would have some discussions and we would be interested in knowing whether this is something that happened before in this indication or for this product. I don’t think we would necessarily, by just observing a difference, say that it’s real, because we know there’s going to many subgroup analyses performed, and there’s going to be random highs and random lows.

Janet Wittes: I see this in companies that list many subgroups and then say they were pre-specified. That’s not pre-specification. If you’re seriously specifying subgroups or secondary outcomes, or whatever, then in the SAP you ought to be able not only to say what direction of effect you expect, but you ought to be able to give some power calculation, and that power calculation should give some formal idea of what your hypothesis really is. You should not simply list a bunch of things and then call them pre-specified.

Kit Roes: Yes. It’s a point I try to make in terms of what’s in the guideline. It’s now focused on predefining the biological rationale. It’s not pre-specifying a list of subgroup tests, but it’s pre-specifying the whole game. What is your rationale for investigating; why you are interested? What’s the biological rationale and how are you intending to analyze the data? And indeed, it doesn’t necessarily need to be type 1 error controlled, but it needs to be fully pre-specified.

Janet Wittes: And you may even say that you don’t expect statistical significance because your sample size is so small. But if you do specify why you’ve made the hypothesis, describe how you are going to test it, your hypothesis becomes much more credible.

Kit Roes: And you won’t do 10,000 hypotheses.

Tom Fleming: Indeed. I’ve argued if a trial is designed to have a dozen secondary endpoints then, in essence, it doesn’t have any. To be viewed as potentially providing “confirmatory” evidence, there should be no more than three or four secondary endpoints/analyses, and these should be carefully selected to provide important complementary insights to those provided by the primary analysis of the primary endpoint. So, Dr Lewis, while several of us have consistently emphasized the importance of pre-specification, reliable evidence about effect modification would not be obtained by conducting two dozen subgroup analyses corresponding to a dozen covariates pre-specified in the SAP.

Hence, it matters when we say “pre-specification” as to its nature. Pre-specifying an intention to conduct many subgroup analyses enables obtaining useful descriptive or “hypothesis-generation” insights about generalizability of treatment effects. However, inclusion of subgroup analyses in primary or secondary alpha-spending analyses could provide confirmatory inferential insights with interpretable *p*-values. The SAP should be clearly written to properly address this distinction.

Janet Wittes: I’d like to add more related to what Dr Kent said. We can avoid these one-subgroup-at-a time analyses easily by adjusting for everything else. So, it’s not males versus females. It’s adjusted for all the other variables. Otherwise, even when we do these quote “pre-specified” analyses, we’re still analyzing one variable at a time.

Lisa McShane: I can’t help but bring up another point regarding pre-specification, and this applies particularly to high-dimensional predictors. One of the most frequent issues we have when we review proposals to evaluate these predictors using specimens that were collected in our NCI trials groups, is the simple question of: can you tell me what the predictor is? How do you calculate it? And that stops a large percentage of people dead in their tracks because they don’t really appreciate what complete specification of a complex predictor means. And this can even happen for single biomarker assays.

Several issues like: are you going to measure this on fresh frozen tumor tissue; or are you going to use archival formalin-fixed paraffin-embedded tissue; or are you going to measure it in plasma versus serum? And oh, by the way, what antibody were you going to use for your immunohistochemical test? It’s amazing how often these things have not been nailed down. And so part of our review process is to pre-specify these aspects.

We encounter some high-dimensional data predictors that are represented only as a list of genes. Well, you have to take a lot more steps to go from that list of genes to actually get some kind of output from a predictor. These are very common problems. And given the heterogeneity with some of these biomarkers, you can see that it’s really important to pay attention to this. You can go from one version of a biomarker assay to another, and what was significant using one could completely disappear in the other. So, these are really critical parts of pre-specification.

Jim Lewis: Dr Wittes, you brought up the issue of power and there was a highly endorsed question about how you respond to the request for post hoc power calculations for these subgroup analyses.

Janet Wittes: I don’t think they make sense. I think you specify the power upfront. How large do I think this subgroup is going to be? What is the power to detect a reasonable effect size? I don’t pay much attention to post hoc power.

Jim Lewis: And if you’re going to do it, you still have to take into account the multiple testing part.

Janet Wittes: Yes.

Lisa McShane: I agree totally with Dr Wittes. But I just want to go back to something she said earlier about the value of doing that power calculation. We often do require power calculation in advance if somebody wants to use specimens from our trials. One might ask, why do I have to do this? The specimens are what they are and we have the number we have. What does it matter? One of the best reasons I have found for requiring the power calculations is to make sure that investigators specify what it is they’re testing, because you can’t talk about power without saying what it is you’re testing.

Jim Lewis: There’s a question here that goes back to a combination of several of the presentations, particularly those of Drs Kent and McShane, about subgroup analysis on a continuous scale. Are there good examples of where somebody has done this that we can use as a guidance of how to proceed? And actually, it even ties into Dr Roes comment earlier, if your cancer proliferation marker KI-67 is 0.1 higher than somebody else, are you really in a different group? So, how do we operationalize this idea of doing this on a continuous scale?

Lisa McShane: I’m not sure that hypothesis testing is quite the way to go if you really have a continuous scale. What I would like to see is a risk score. That could be a single biomarker. It could be a multi-variable risk score. Give me confidence intervals on a plot that shows a curve with outcome on the Y-axis, maybe 5-year disease-free survival, and represents the continuous score on the X-axis. What I often find is that for practical reasons people will use dichotomization for purposes of a quick and easy power calculation, because it’s not so easy to determine what sample size you need to get tight bounds on a risk curve when that risk curve may be quite complex and require many assumptions that you cannot completely specify up front.

Tom Fleming: That’s really important, Dr McShane, because if there is effect modification on a continuous covariate, it’s highly plausible that it’s not a truly dichotomous threshold effect. Proposals by Dr McShane and others to consider effect modification on a continuous scale are wise. The problem, of course, is having sufficient evidence to be able to understand what that true functional relationship is.

Lisa McShane: That’s right.

Janet Wittes: I think it’s also interesting to ask whether, if you have a continuous covariate, why put them in quartiles or quintiles? Why not just use the continuous variable itself?

Lisa McShane: Again, I think it gets back to modeling. How easy is it to model what could be a complex shape. I would agree, if you can do it, continuous is the way to go. It gets back also to what the clinician wants. In the end, while everyone likes to have their cut points for clinical decision-making because they think it gives them a clean yes or no answer, it doesn’t really give the clinician a clean yes or no answer. They’d be better off if they had a risk curve and they had the continuous value so they can say, well, Ms Jones is 0.001 below the cut point, but if she had been that much higher, she would have gotten the treatment.

But then you say, well, okay, Ms Jones has these other clinical characteristics (maybe comorbidities), and factor all of that into the picture. So, I think that is something that we as a medical community should really push more for—giving those continuous values in a report, even if you might ultimately decide to follow the usual clinical guideline, say that if you’re above 10 on the cancer tumor mutation burden, then you get immunotherapy. But I think having that value is important, and especially in light of the variability across assays. Most hospitals or health systems will use the assays that they have up and running in their lab, regardless of what FDA has approved as a companion diagnostic. That is the reality. I’m not saying that’s a good thing, but that is the reality.

Jim Lewis: I’m going to ask one last question from the audience, and then I’m going to ask each of you in 30 s or less to give what you think was the most important take-home message from this morning. The question from the audience is, we’ve heard several examples of trials that showed a negative overall result, but a positive result in a subgroup that failed to be replicated in a follow-up trial. Is anyone aware of a case in which the subgroup analysis was confirmed in a follow-up trial? Has there been a systematic review of this idea that people could read?

David Kent: I can provide an example. They tested activated protein C for sepsis in the PROWESS Trial.^13^ They found it worked overall; it had a strong effect reducing mortality. Subsequently, I think it was the FDA that required a risk-based analysis. It turned out that in the high-risk subgroup with an ICU mortality APACHE score greater than 25 it worked very well, like twice the effect seen in the overall group. But in those with an APACHE score of 25 or less, it worked not at all, and it caused a lot of bleeding.

But I guess the statisticians at the company knew that they should be skeptical of subgroups. So they said, we think that’s a spurious result; we want to do this again. So, they just took the low-risk patients with an APACHE score of 25 or less and they ran a second trial, the ADDRESS Study.^14^ It didn’t work at all to reduce mortality—it just caused a lot of bleeding. So, that’s just one example, but I’m sure there are many.

Jim Lewis: Dr Wittes, if you could tell us what do you think is the most important take-home from this morning?

Janet Wittes: It depends on who you are that determines the most important take-home. For me, the most important take-home is that we will continue to do subgroup analyses of one kind or another. But we must be very cautious about interpreting them and, if we can, we need to see if the findings can be replicated.

Lisa McShane: It’s hard to beat Janet’s answer. I think replication is the key but realizing that it may be hard to do that second trial, because people, especially when it comes to biomarkers, believe the results. They think biomarkers are magic. Some would feel that if you’re not sure if there’s really a benefit in that negative group, why would I want to go on that trial, especially in oncology when you can often get drugs off label. So yes, I think replication is the answer and also pre-specifying as much as you can, giving that biological rationale up front. I’ve had the same experience as many where you produce a result and you can get a post hoc explanation for any result you hand your collaborator.

Tom Fleming: The most important take home from this morning when defining criteria for credibility of results in subgroups is that these results should be statistically compelling and biologically plausible. And for both of those criteria, pre-specification is critical, and where replication often is key for results to be compelling.

When conducting exploratory analyses in search of positive results, it also is important to keep in mind that “random high bias” is not an artificial construct. It’s real, that is, effect sizes in subgroups with the most favorable estimates will tend to be overestimates of truth.

Finally, coming back to Lisa McShane’s presentation—if the biomarker negative patients are excluded when assessing the effects in the biomarker positives, then it is illogical to allow the positives to drive the analysis addressing whether benefit in the positive could be extrapolated to the negatives. The Rothmann et al.^12^ paper provides a proper approach to address this extrapolation.

David Kent: To avoid redundancy, I’ll focus on risk. But I agree with what other people have said in terms of credibility of subgroup analyses and the extreme need for caution. As a clinician, I really want to know what the effects of a treatment are across the whole spectrum of risk, and I can’t see that if you’re just presenting me the overall results. I can’t see that if you’re just showing me the usual one-variable-at-a-time subgroup analyses. I can only see that when you dis-segregate the patients by risk using a multi-variable prediction tool. That’s going to give me a lot more information.

Ellis Unger: I think given all the issues here and all the complexities, if you want to make something out of a subgroup analysis from a registrational trial, it probably behooves you to talk to the regulators, and not just slide something quietly into the SAP. You should explain it to the regulators when you’re planning your trial. Talk to the European Medicines Agency and FDA, and say, “Look, we plan this subgroup analysis. This is our reasoning and our plan. What do you think?” That’s my advice based on the proceedings this morning.

Kit Roes: Last in the row, so very difficult to avoid duplication, because I think most good points have been raised. I agree that we are moving towards a common understanding of the challenges of subgroups, but we might have to get our stakeholders onboard. We have a very important role in communicating with non-statisticians on the issues of subgroup analysis. We are at the verge of convincing ourselves on most of the challenges and the way to move ahead, but the real challenge is probably in educating the non-statisticians on how they should view subgroup analysis.

Jim Lewis: Well, I want to say it has truly been an honor for me to spend the morning with the 6 of you and 420 other people. On behalf of the audience and the organizers, we cannot thank you enough for providing such incredible education to all of us today.

## Participants

Tom Fleming, PhD, University of Washington

David Kent, MD, Tufts University

James Lewis, MD, University of Pennsylvania

Lisa McShane, PhD, National Cancer Institute

Kit Roes, PhD, Radboud University Nijmegen Medical Centre

Ellis Unger, MD, Hyman, Phelps & McNamara, P.C.

Janet Wittes, PhD, Wittes, LLC
